# An Innovative Approach of Utilizing the Digastric Tendon for Lower Lip Soft Tissue Repair: A Case Report

**DOI:** 10.7759/cureus.69320

**Published:** 2024-09-13

**Authors:** Mohammed Azeem Khan, Firoz Borle, Chandrashekhar Mahakalkar, Shivani Kshirsagar, Sparsh Dixit, Pankaj Katariya

**Affiliations:** 1 Department of Surgery, Jawaharlal Nehru Medical College, Datta Meghe Institute of Higher Education and Research, Wardha, IND

**Keywords:** digastric tendon, fibula flap, lower lip, reconstruction, tissue repair

## Abstract

Lower lip reconstruction following oral and neck oncosurgery presents significant challenges in maintaining function and esthetics. This case report describes a novel application of the digastric tendon for repairing the soft tissue of the lower lip in a patient undergoing wide excision of a lesion, bilateral modified radical neck dissection, segmental mandibulectomy, and free fibula flap repair. This innovative approach aims to maintain oral competence, strengthen the flap, and enhance esthetics. The digastric tendon was chosen due to its accessibility and effectiveness in strengthening the lower lip.

## Introduction

Advanced oral cancer often necessitates wide excisions that involve the removal of several structures, such as the tongue, lip, external skin, oral mucosa, and mandibular bone [1]. Addressing significant structural and functional defects from these resections requires complex reconstructions to restore mandibular skeletal defects, generate adequate oral lining, cover enough skin, and maintain swallowing mechanisms [2]. Esthetic considerations also play a crucial role in promoting social interaction [3].

For head and neck reconstructions in the past, pedicled flaps like the latissimus dorsi and pectoralis major musculocutaneous flaps have been used [4]. However, these flaps often fall short of providing the essential tissue components required for bony reconstruction. Flap prefabrication, while applicable to some patients, usually yields thin flaps unsuitable for composite reconstruction [5]. Free tissue transfer, particularly through microsurgery, has become the standard approach for addressing significant head and neck defects. Due to several advantages, the free fibula osteocutaneous flap is increasingly preferred over the iliac crest flap. Research indicates that the fibula flap offers a stable skin island, which is crucial for successful reconstruction, alongside a long vascular pedicle that enhances the blood supply and viability of the tissue [6]. Additionally, the abundant bone supply from the fibula allows for the effective reconstruction of osseous defects, making it a versatile option in complex cases [7]. While the iliac crest flap has traditionally been used, its limitations in terms of donor site morbidity and variability in bone quality have led to a shift in preference toward the fibula flap [8,9].

Despite advancements, functional results following complex reconstructions vary, especially regarding oral competence. Techniques that support oral competence in complete oromandibular reconstructions have been underexplored. This case report highlights the innovative use of the digastric tendon for reconstructing extensive lower lip defects, demonstrating favorable outcomes and emphasizing its potential as a valuable adjunct in complex reconstructive procedures. Utilizing the digastric tendon technique in lower lip reconstruction presents several significant benefits. By integrating this methodology, surgeons can achieve enhanced functional and esthetic results such as improved mobility and facial expressiveness [10]. The inherent properties of the tendon serve to meet the structural and dynamic needs of the lower lip, thereby promoting a more robust and efficient reparative process. The digastric tendon technique offers significant advantages in enhancing support and functionality, particularly in surgical contexts where traditional methods may be inadequate.

## Case presentation

**Figure 1:**
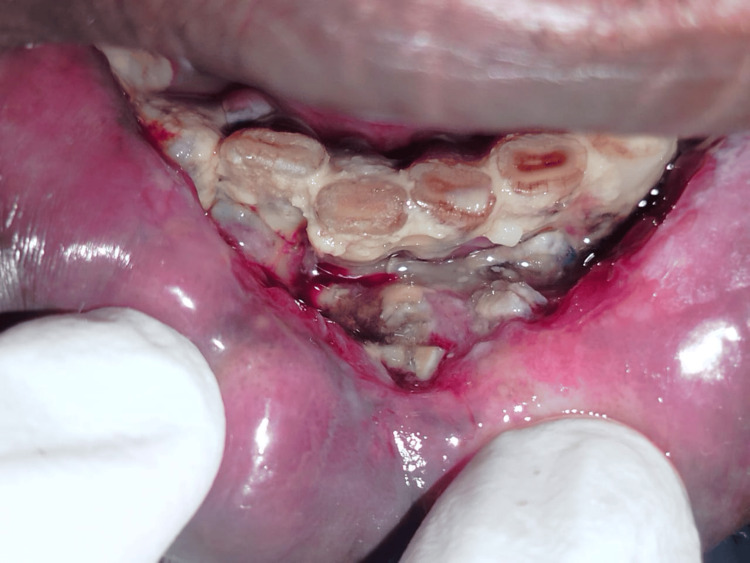
Intraoral view of a lower mandible lesion diagnosed as squamous cell carcinoma, with visible bone exposure and necrosis. Surrounding tissues appear erythematous and inflamed

A 55-year-old man with a history of tobacco use presented with a nonhealing ulcer in the right lower mandibular region, which had gradually increased in size over one year (Figure 1). Squamous cell carcinoma was diagnosed following a biopsy. Imaging studies revealed a clinical stage of cT4aN2bM0. The patient underwent subtotal mandibulectomy and bilateral modified radical neck dissection, performed by an oral surgeon, with simultaneous reconstruction by our plastic surgery team.

**Figure 2:**
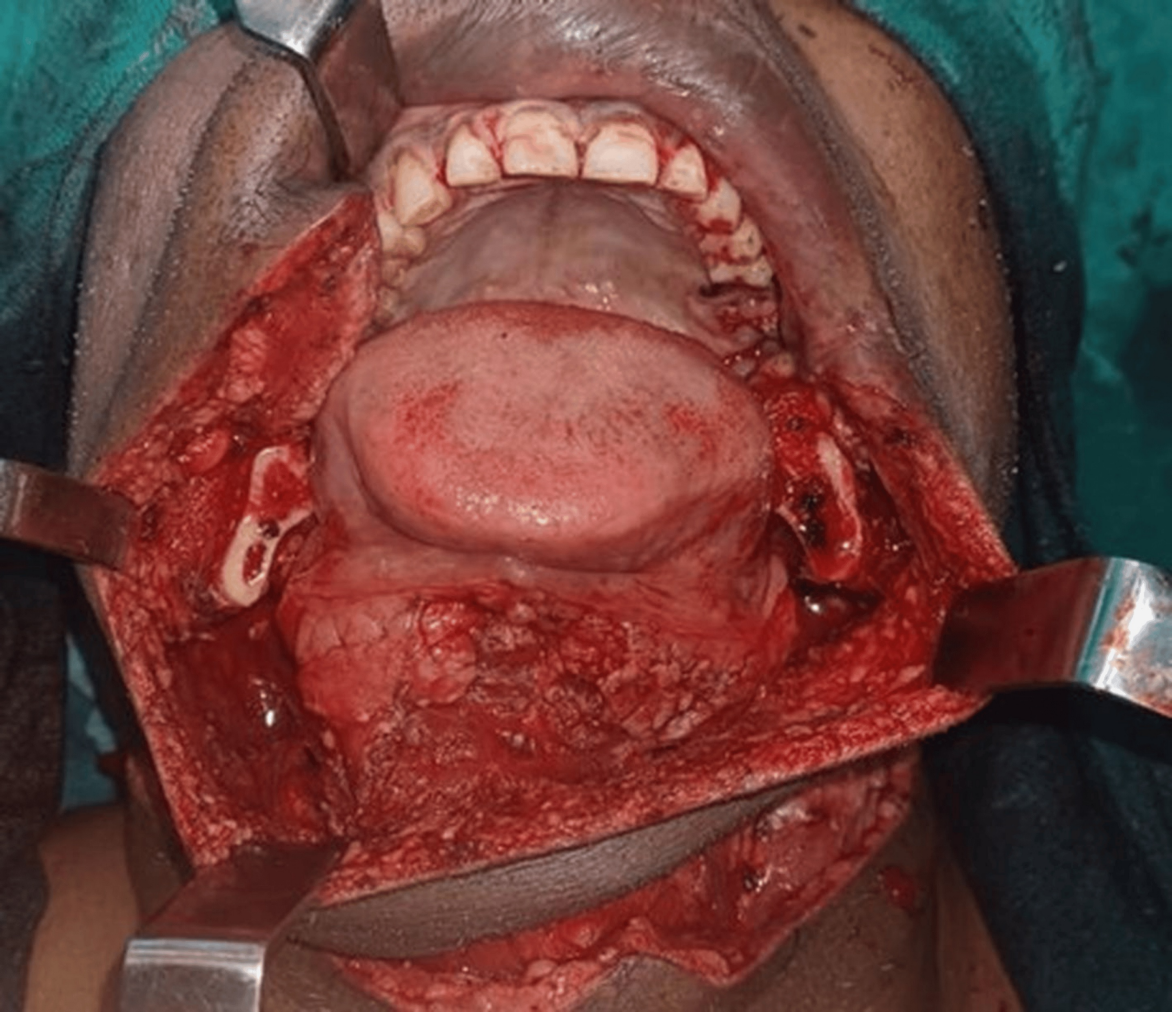
Intraoperative view of the surgical defect, exposing the lower jaw and tongue. Multiple retractors are in place to provide clear access to the surgical field, which includes the oral cavity and surrounding tissues

A segmental mandibulectomy was performed from the right mandibular second premolar (tooth 36) to the angle of the mandible, achieving a surgical margin of at least 1 cm from the tumor edge (Figure 2). In a bilateral modified radical neck dissection, the facial arteries were resected while the lymph nodes from levels I to V were removed, sparing the internal jugular vein, sternocleidomastoid muscle, and spinal accessory nerve.

**Figure 3:**
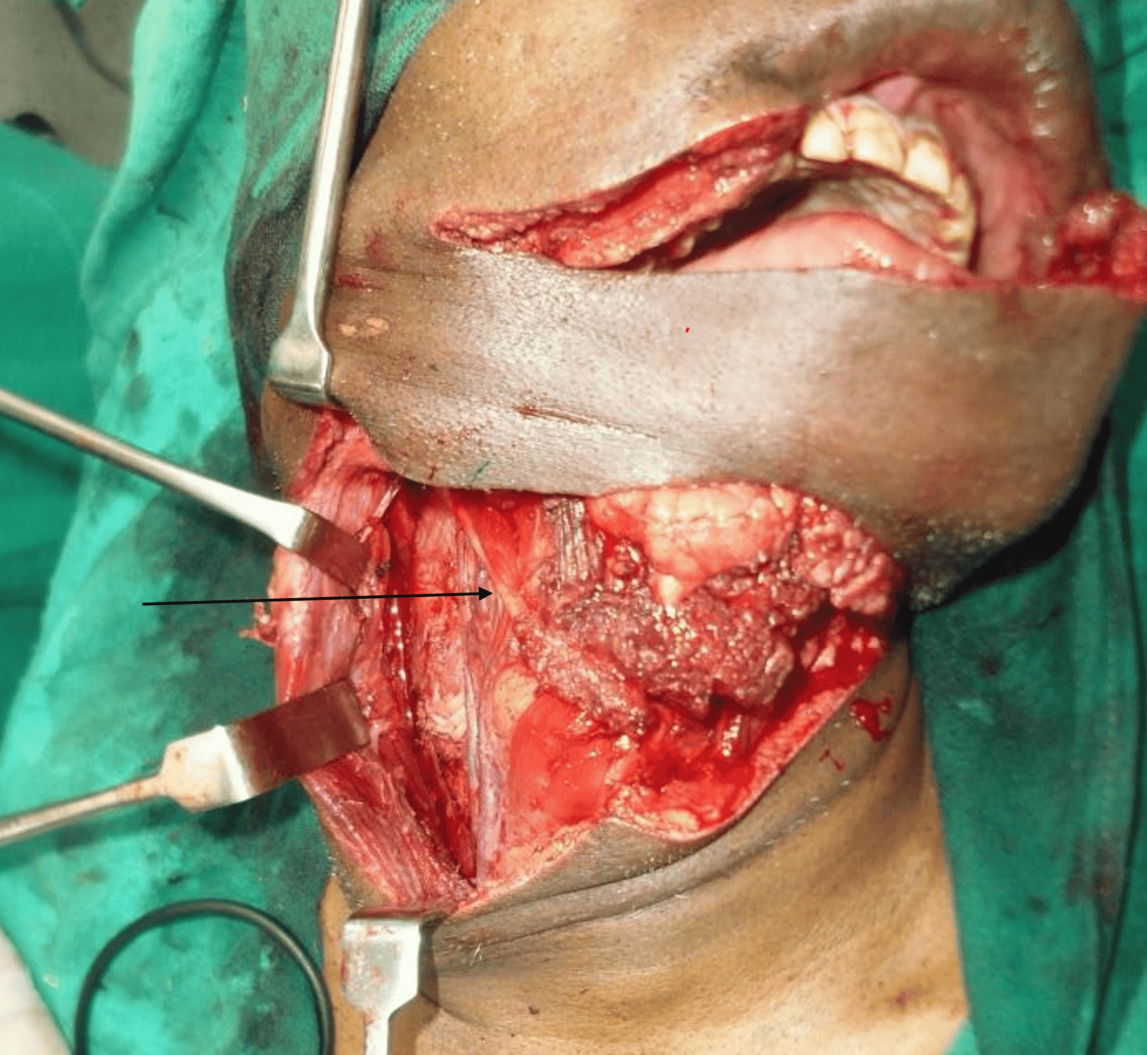
Intraoperative photograph demonstrating the identification of the digastric muscle, emphasizing meticulous muscle dissection necessary to prevent damage to adjacent tissues from its attachment to the hyoid bone

**Figure 4:**
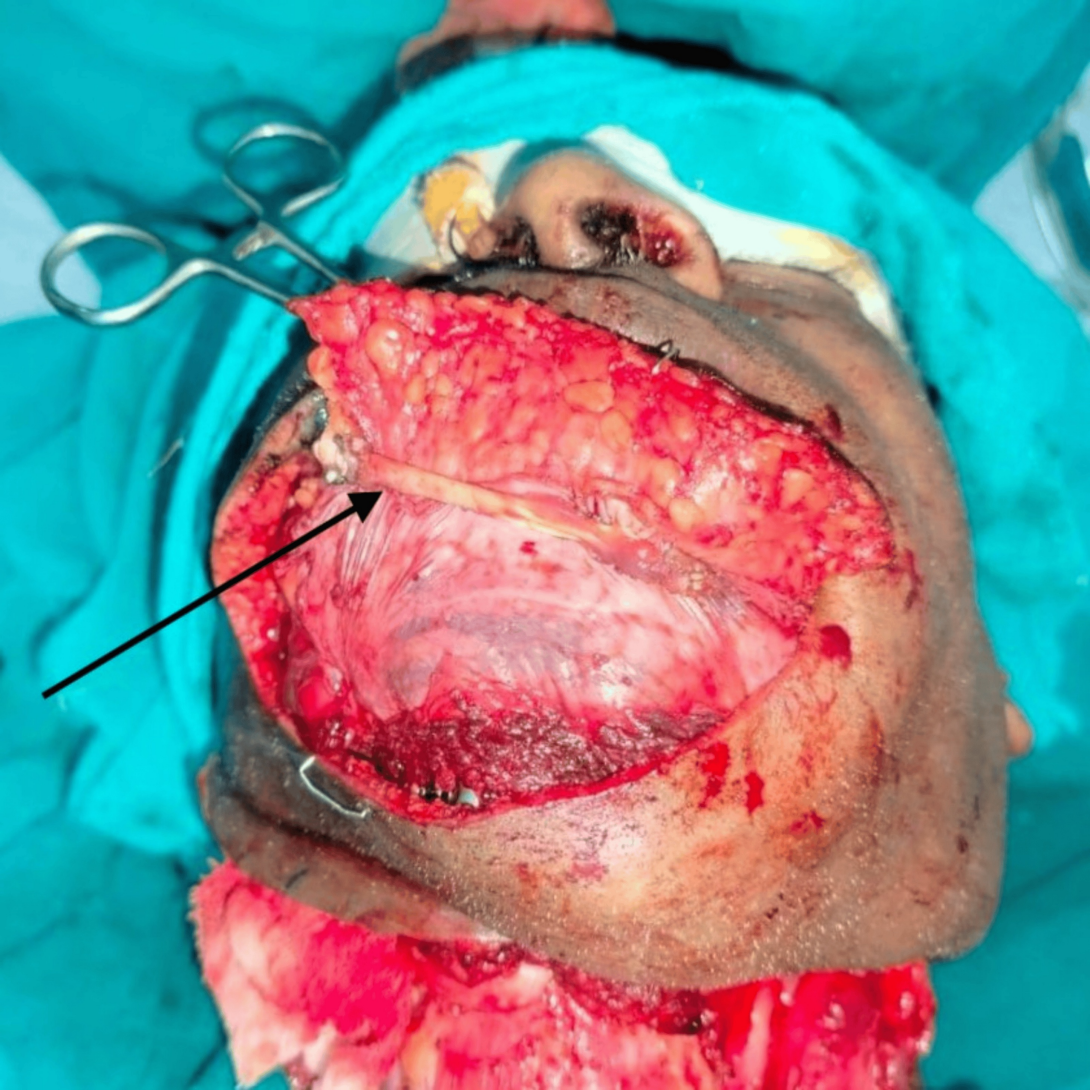
Lower lip reconstruction using digastric tendon anchoring. The intraoperative view shows the exposed surgical site with the arrow indicating the point of attachment between the remnant orbicularis oris muscle and the digastric tendon

A free fibula flap, comprising a portion of the fibula bone, the skin paddle above it, and the vascular pedicle (peroneal vessels), was lifted from the left leg by our plastic surgery team. The flap was contoured to match the resected mandibular segment and fixated with titanium plates and screws. Microvascular anastomosis connected the flap artery and great saphenous vein graft to the right facial artery and the right external jugular vein using 9-0 nonabsorbable sutures. For soft tissue reconstruction of the lower lip, the right digastric tendon was used to connect the remnant orbicularis of the lower lip, as it was free of cancer cells and easily accessible after neck dissection. The digastric tendon was carefully identified and dissected from its attachment at the hyoid bone (Figure 3). The tendon was then incised near its insertion and meticulously mobilized to provide adequate length for transfer. Next, the tendon was routed superiorly and laterally, securing it to the orbicularis oris muscle with nonabsorbable sutures to restore lower lip competence and symmetry. Precise tension was maintained to ensure functional restoration without excessive strain on the tendon. To determine the optimal lower lip positioning, temporary sutures were placed intraoperatively to approximate the desired outcome. Despite the muscle paralysis induced by anesthesia, using these sutures and careful visual and tactile assessment allowed for accurate positioning of the digastric tendon transfer. The final adjustments were confirmed by manually simulating the tension exerted by the orbicularis oris during natural facial movements. The tendon was sutured with 3-0 nonabsorbable sutures after being routed through a tunnel made in the upper lip's orbicularis oris muscle, facilitating functional contraction transmission to the reconstructed lower lip (Figure 4). The membranous fascia lata in the folded fibula flap was sutured to the surrounding facial muscle stumps with 3-0 nonabsorbable sutures.

**Figure 5:**
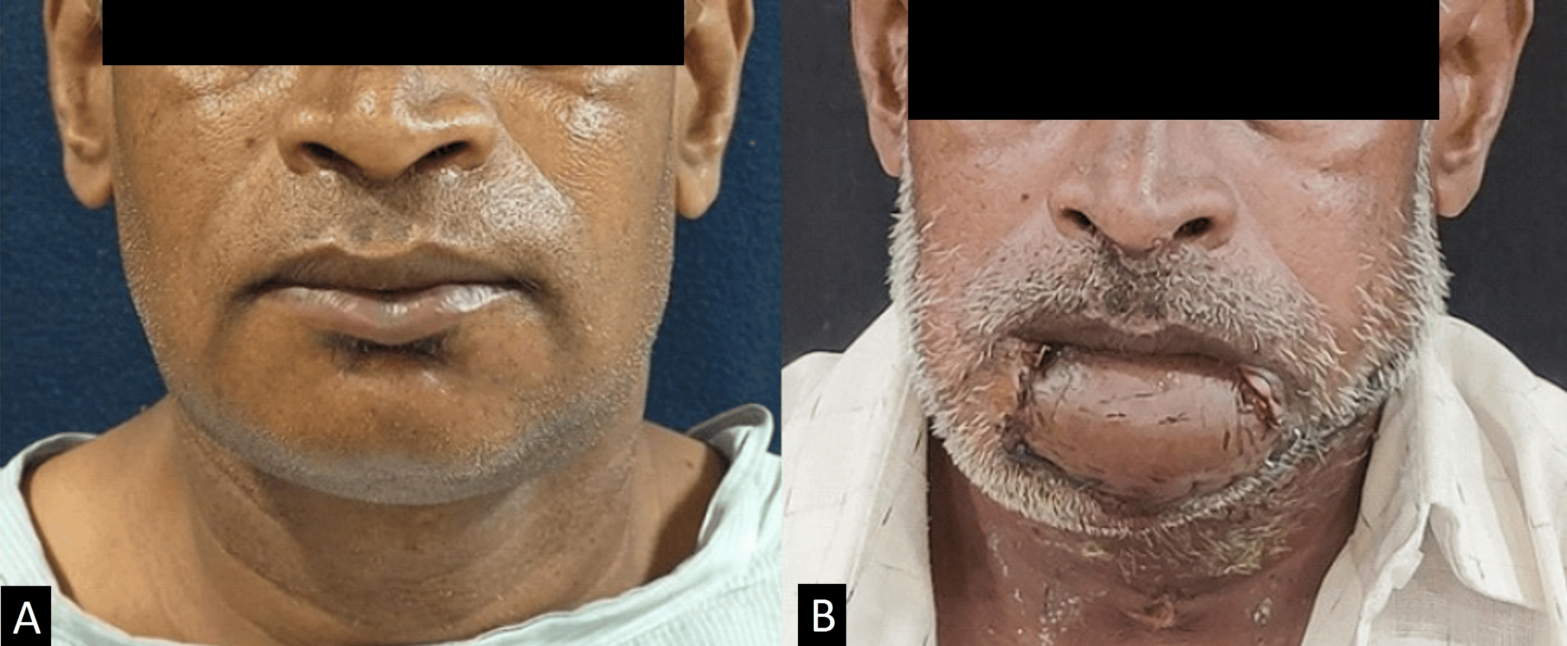
Comparison of the (A) preoperative frontal view of the lower lip before surgery and (B) postoperative frontal view, demonstrating the result after tumor excision and soft tissue repair using the digastric tendon anchoring technique. The restoration of lip contour and function despite significant tissue loss is visible

The total operative time was approximately 12 hours. The patient was monitored in the intensive care unit postoperatively for flap viability with regular Doppler ultrasound assessments and received prophylactic antibiotics and anticoagulation therapy to prevent flap thrombosis. Postoperatively, on follow-up, the patient demonstrated significant improvement in oral competence and considerable restoration of lip contour and function despite substantial tissue loss, evidenced by the ability to close the mouth without drooling (Figure 5).

## Discussion

The application of the digastric tendon for lower lip reconstruction in extensive oral and maxillofacial surgery presents a potential technique. Restoring function and achieving esthetically pleasing results are critical in complex reconstructions involving multiple structures [2]. The significant functional benefit of the digastric tendon is the marked improvement in oral competence. Anchoring the tendon to the remnant orbicularis oris muscle provided essential support to the lower lip, reducing sagging and preventing drooling. Better control over oral secretions, which are essential for speaking and swallowing, was made possible by this approach. Digastric muscle transfer has been shown to be useful in face reanimation, especially when there is facial nerve palsy. It aids in the restoration of dynamic movement and symmetry [10].

In terms of appearance, the lower lip's natural shape was restored by using the digastric tendon. In reconstructive surgery, achieving both practical and esthetic goals is essential. The patient's self-esteem and satisfaction with the surgical outcome rose as a result of the better symmetry and decreased lower lip drooping. One of the most important surgical factors to take into account is tendon viability. It was vital to maintain the digastric tendon's blood supply. Careful surgical technique made sure that the incorporation into the surrounding tissues went well. In order to prevent either an under or overcorrection, proper tension adjustment was necessary. By balancing the functional and cosmetic results, intraoperative changes enabled the lower lip to be positioned optimally. Minimizing extra morbidity using the digastric tendon, which is accessible after neck dissection, decreased the amount of time needed for surgery and limited extra morbidity, making it a useful technique for intricate reconstructions [11].

While proven efficient, traditional lower lip replacement methods such free tissue transfer, local flaps, and muscle slings have drawbacks like prolonged recovery periods and donor site morbidity [12]. As a complementary technique, the use of digastric tendon leverages existing surgical sites to streamline the procedure and reduce patient strain. For patients requiring substantial soft tissue support, this approach enhances the free flap procedure by providing additional support and stability, balancing ease of implementation with effective outcomes. By integrating the digastric tendon approach, surgeons can optimize the reconstruction process while minimizing the need for additional incisions and potential complications. Research highlights that novel approaches to head and neck reconstruction can result in better functional and morphological outcomes. Studies have emphasized the significance of establishing oral competency and esthetic symmetry in patients with extensive resections [13]. The digastric muscle is useful in improving oral competency and attractiveness in lower lip reconstructions because of its function in face reanimation. Combining this technique with other reconstructive methods may offer insights into optimizing outcomes for complex oromandibular reconstructions.

## Conclusions

Using the digastric tendon for lower lip reconstruction has shown favorable results, particularly in enhancing oral competence and reducing drooling. While research suggests that the digastric sling, when used in conjunction with flap techniques, can significantly improve outcomes by decreasing drooling and supporting dynamic reanimation of the lower lip, these findings are still preliminary and based on limited cases. The digastric muscle's role in restoring symmetry and movement and reducing sagging is encouraging, with patients reporting high satisfaction and minimal complications. However, despite the functional and esthetic improvements noted in these early cases, further research involving larger cohorts is needed to validate the efficacy and safety of this approach. In conclusion, while the digastric tendon transfer shows potential as a viable option for lower lip reconstruction, these results should be interpreted with caution. Additional studies are essential to confirm its applicability and ensure consistent outcomes in more complex reconstructive surgeries.
